# Preparation of Advanced CuO Nanowires/Functionalized Graphene Composite Anode Material for Lithium Ion Batteries

**DOI:** 10.3390/ma10010072

**Published:** 2017-01-17

**Authors:** Jin Zhang, Beibei Wang, Jiachen Zhou, Ruoyu Xia, Yingli Chu, Jia Huang

**Affiliations:** School of Materials Science and Engineering, Tongji University, Shanghai, 201804, China; 1433112@tongji.edu.cn (J.Z.); 1433037@tongji.edu.cn (B.W.); 1531468@tongji.edu.cn (J.Z.); 1531466@tongji.edu.cn (R.X.); 11chuyingli@tongji.edu.cn (Y.C.)

**Keywords:** functionalized graphene, one-pot synthesis, CuO, lithium-ion battery, electrochemical performance

## Abstract

The copper oxide (CuO) nanowires/functionalized graphene (f-graphene) composite material was successfully composed by a one-pot synthesis method. The f-graphene synthesized through the Birch reduction chemistry method was modified with functional group “–(CH_2_)_5_COOH”, and the CuO nanowires (NWs) were well dispersed in the f-graphene sheets. When used as anode materials in lithium-ion batteries, the composite exhibited good cyclic stability and decent specific capacity of 677 mA·h·g^−1^ after 50 cycles. CuO NWs can enhance the lithium-ion storage of the composites while the f-graphene effectively resists the volume expansion of the CuO NWs during the galvanostatic charge/discharge cyclic process, and provide a conductive paths for charge transportation. The good electrochemical performance of the synthesized CuO/f-graphene composite suggests great potential of the composite materials for lithium-ion batteries anodes.

## 1. Introduction

Nowadays, lithium ion batteries (LIBs) have become one of the primary power sources due to their excellent advantages in capacity, cyclical stability, and sustainability. Therefore, they have been widely applied in telephones, personal computers, and electric vehicles [1,2,3,4,5]. Transition-metal oxides (TMO), such as TiO_2_, MnO, SnO_2_, CuO, and Fe_2_O_3_, have been researched as anode materials for LIBs for several decades due to their high theoretical capacity and environmental benignity [6,7,8,9,10]. In addition, ternary oxides have also been widely studied by scientific researchers [11]. For instance, Reddy et al. prepared spinel MCo_2_O_4_ (M = Mg, Mn) materials with high capacities via the molten salt method [12]. Among them, copper oxide (CuO) is a nontoxic and abundant material which is cheap and has higher theoretical capacity than graphene and carbon black [13,14]. These features suggest it is suitable for the anode material in LIBs [15,16,17]. However, just like the other TMO-based anode materials, CuO displays large volume expansion (174%) and particle pulverization during the cyclic charge/discharge process [18,19]. Additionally, CuO has a low electrical conductivity (p-type semiconductor), and many studies have been done to improve it by adding conductive carbon materials, such as graphene, carbon nanotubes (CNTs), fullerene, and so on. Graphene, a two-dimensional (2D) material of carbon just one atom thick, is a remarkable support material for active nanomaterials due to its strengths in high electrical conductivity, thermal conductivity, flexibility, large surface area, and chemical stability [20,21,22]. When incorporated with TMO nanoparticles, the graphene sheet can sustain, induce the nucleation, growth, and uniform dispersion, and also restrain the volume expansion of them during cyclic testing, and the nanoparticles, in return, will prevent the graphene sheet from agglomerating and increase the surface areas of them. Thus, it is expected that the composite will have a better electrochemical performance than its individual counterparts.

Recently, CuO and graphene composites with various morphologies have been fabricated by using different synthetic processes [23,24,25,26,27]. Zhang et al. have synthesized a novel porous CuO nanorod/reduced-graphene oxide (rGO) composite via a solvothermal method [28], Liu et al. successfully prepared flexible CuO nanosheets/rGO composite paper through the vacuum filtration and hydrothermal reduction method [29], and Zhou et al. have fabricated CuO hollow nanoparticles/graphene-nanosheet composites via the Kirkendall-effect approach [30].

In most of these studies, rGO sheets were used. The rGO sheets were obtained by oxidizing graphene into graphene oxide first, and reducing it in the following step. However, the oxidation step will always damage the mircrostructure and molecular structure of graphene, and the reduction process could not completely restore its electrical conductivity behaviour [31]. Here in this work, we have desigened a novel, easy, and quick method to synthesize the CuO/functionalized graphene (f-graphene) composite by a one-pot wet chemistry method, which is reported for the first time, to our best knowledge. “–(CH_2_)_5_COOH” groups were introduced onto f-graphene sheets via the Birch reduction chemistry method, which function as the nucleation sites for the formation of CuO nanowires (NWs) without adding any surfactants, according to previous literature [32]. This method can produce graphene sheets with less defect damages and better electrical conductivity than rGO. The as-synthesized CuO NWs can be well dispersed on the f-graphene sheets. The f-graphene effectively restrains the volume expansion of CuO during the cyclic charge/discharge process while maintaining its great flexibility and high electrical conductivity. When used as anode materials in LIBs, the CuO/f-graphene composite shows superior electrochemical stability than the pure CuO material, and the specific capacity is higher than other CuO/graphene composites reported before [23,25,28,30].

## 2. Experimental

### 2.1. Synthesis of CuO/f-Graphene Composite

**Scheme 1:**
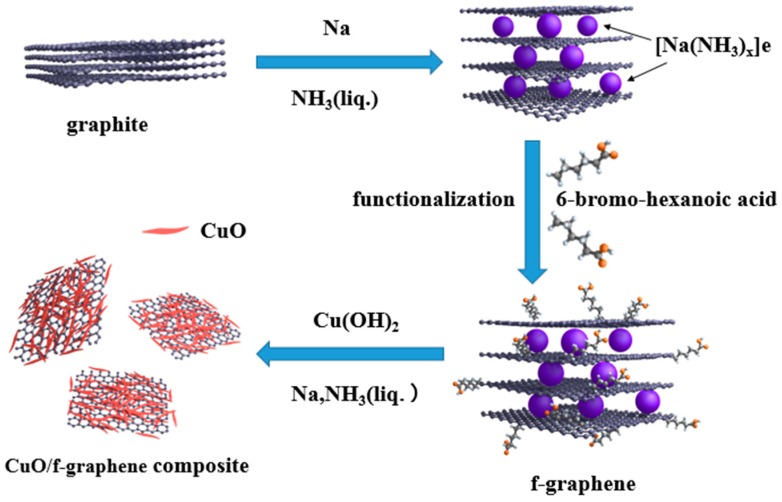
Schematic of the synthesis procedure of the CuO/f-graphene composite.

The CuO/f-graphene composite was successfully synthesized via a one-pot chemistry method. The f-graphene was prepared through the Birch reduction chemistry method to yield “–(CH_2_)_5_COOH” groups and the detailed processes were shown as follows [32]: firstly, liquid ammonia (70 mL) was cooled with the mixture of ethanol (95%, Greagent, Chengdu, China) and dry ice in a 250 mL flask. Then graphite (50 mg) and sodium (145 mg, Acros, Shanghai, China, 98.8%) were added into it. After continuous stirring for 20 min, 6-bromo-hexanoic acid (1.625 g, Adamas, Shanghai, China, 98%) was added and stirred for about 50 min. To achieve highly functionalized graphene, sodium, and 6-bromo-hexanoic acid were added three times in turn. After that, sodium (200 mg) was added into the flask to react for 10 min before Cu(OH)_2_ (105 mg, Greagent, Chengdu, China) was added and reacted for 50 min. The mixed solution was stirred overnight. After the liquid ammonia evaporated completely, the product in the flask was transferred and mixed with hexane, and then washed by NaOH solution (pH = 11) three times to remove impurities. Finally, the CuO/f-graphene composite was dried at 60 °C overnight. The schematic of the synthesis procedure of the CuO/f-graphene composite is depicted in Scheme 1. For comparison, the f-graphene sample was synthesized without adding Cu(OH)_2_, and the pure CuO material was synthesized by the same method without adding graphene.

### 2.2. Materials Characterizations

The morphology of CuO NWs were characterized by using the field-emission scanning electron microscope (FESEM, FEI, QUANTA250FEG, Brno, Czech Republic) at 30 kV. Transmission electron microscopy (TEM) and high-resolution transmission electron microscopy (HRTEM) were measured on a JEOL JEM-2100F TEM at 200 kV (JEOL, Tokyo, Japan). The crystalline phases of the composite were studied by an X-ray diffractomer (XRD, Dandong Haoyuan Instrument Co. Ltd., Dandong, China) scanning from 10° to 80° with Cu K_α_ radiation. Thermo-gravimetric analysis (TGA) was tested at a heating rate of 10 °C·min^−1^ in air from room temperature to 800 °C by using a NETZSCH TG-DTA/DSC analyzer (NETZSCH, Selb, Germany). The Raman spectrum was measured on a DXR Raman Microscope (Thermo Fisher Scientific, Waltham, MA, USA) via a 633 nm He-Ne laser.

### 2.3. Electrochemical Measurements

The anode electrodes were made up of the CuO/f-graphene composite, polyvinylidene fluoride (PVDF), and conducting acetylene black with a weight ratio of 80:10:10 dissolved in N-methyl-2-pyrrolidinone (NMP) solvent. Then, the slurry was coated on a Cu foil and heated at 120 °C for 12 h in a vacuum drier. The average mass coated on the electrodes is about 1 mg·cm^−2^. The assembly of coin cells was performed in an argon-filled glove box by using CR 2025 coin-type cells with lithium foil counter-electrodes, Celgard 2400 membranes as separators, and 1.0 M LiPF_6_ in ethyl methyl carbonate (EMC)/ethylene carbonate (EC)/dimethyl carbonate (DMC) (volume ratio 1:1:1) as electrolyte. The galvanostatic charge/discharge performances were measured on a multichannel battery tester (LANHE, LAND 2001A, Wuhan, China) with a voltage range from 0.01 to 3.0 V at room temperature. The cyclic voltammetry (CV) and electrochemical impendence spectroscopy (EIS) measurements were carried out on an electrochemical workstation (Chenhua, CHI660E, Shanghai, China).

## 3. Results and Discussion

**Figure 1:**
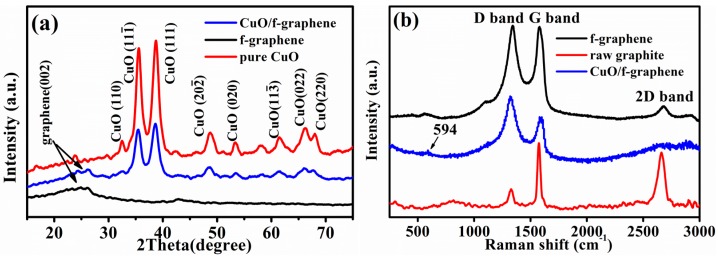
(**a**) XRD patterns of the f-graphene, pure CuO, and the CuO/f-graphene composite; and (**b**) Raman spectra of raw graphite, the f-graphene, and the CuO/f-graphene composite.

Figure 1a represents the XRD patterns of pure CuO powder, the f-graphene and the CuO/f-graphene composite. The as-synthesized CuO has almost the same diffraction peaks compared with the standard CuO phase (JCPDS standard card No. 48-1548). The f-graphene shows a low crystallinity behavior with only the characteristic broad peak at 25°, indexed to the (002) plane, could be clearly observed. Obviously, the diffraction pattern of the CuO/f-graphene composite is the superposition of both CuO and the f-graphene, which indicates the f-graphene sheets have been exfoliated from the graphite effectively and the CuO NWs have been incorporated into the graphene sheets successfully.

The Raman spectrum of f-graphene (Figure 1b) shows two prominent peaks near 1350 cm^−1^ and 1600 cm^−1^. The former one, named D band, reveals the defect of the C atomic lattice while the latter peak called G band represents the stretching vibration of C atom sp^2^ hybrid plane. The intensity ratio (D/G ratio) shows an obvious increase after functionalization. The result suggests that the functional groups “–(CH_2_)_5_COOH” were successfully attached to the graphene and the sp^3^ defect centers were introduced [29,33]. Moreover, the peak at 594 cm^−1^ in the Raman spectrum of the CuO/f-graphene composite is in agreement with the Raman signal of the monoclinic CuO, which gives the CuO NWs a priority to react on these defective positions.

**Figure 2:**
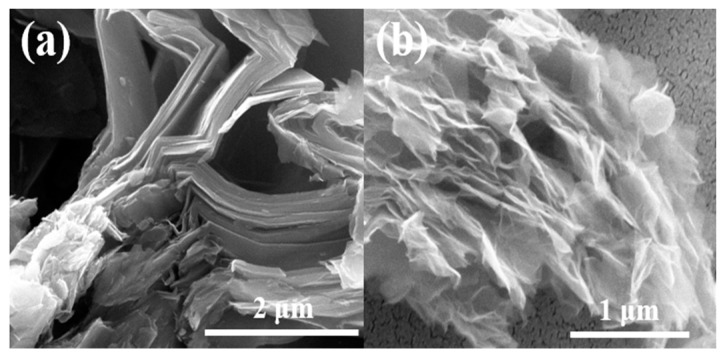

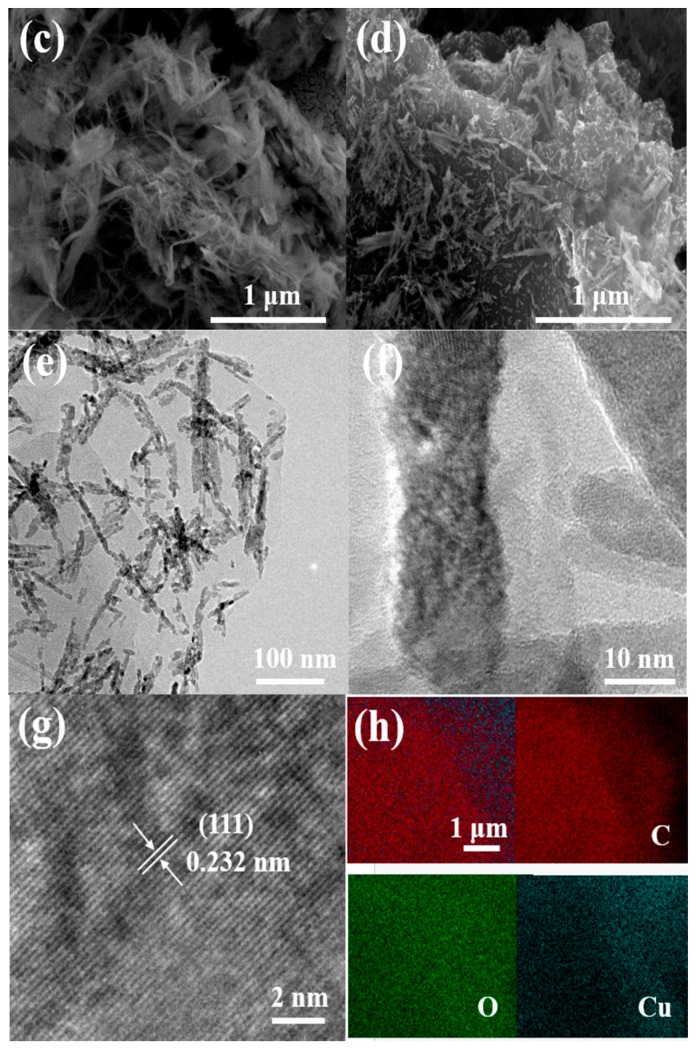
SEM images of (**a**) raw graphite; (**b**) the f-graphene; (**c**) pure CuO NWs; (**d**) the CuO/f-graphene composite; (**e**) TEM image of the CuO/f-graphene composite; (**f**,**g**) HRTEM images of the CuO/f-graphene composite; and (**h**) SEM elemental mapping of the CuO/f-graphene composite.

The SEM, TEM, and HRTEM images of raw graphite, the f-graphene, pure CuO, and the CuO/f-graphene composite are shown in Figure 2. The graphite raw material has a compact lamellar microstructure (see Figure 2a). After functionalization, the f-graphene shows a nanosheet structure (Figure 2b), and it can be clearly observed that the interlayer spacing of graphene nanosheets have increased significantly compared to that of the raw graphite, which leave space for CuO NWs to intercalate into the f-graphene nanosheets. Figure 2c shows the morphology of pure CuO NWs with the length of 300–600 nm and the width of 10–30 nm. Figure 2d shows the f-graphene/CuO composite synthesized via the one-pot method. The CuO NWs are found to be homo-dispersed on the f-graphene nanosheets with 300 nm to 500 nm length without obvious aggregation. Moreover, it conformed to the TEM image of the CuO/f-graphene composite in Figure 2e. Figure 2f is the HRTEM image of individual CuO NW with the width of 10–20 nm. Furthermore, the lattice spacing is 0.232 nm (see Figure 2g), which matches well with the (111) crystalline plane of crystalline CuO [19,34,35]. This result is also consistent with the corresponding XRD pattern of the CuO/f-graphene composite shown in Figure 1a. In Figure 2h, the energy dispersive X-ray analysis (EDX) elemental mappings further proves that the CuO NWs have been evenly dispersed on the f-graphene.

**Figure 3:**
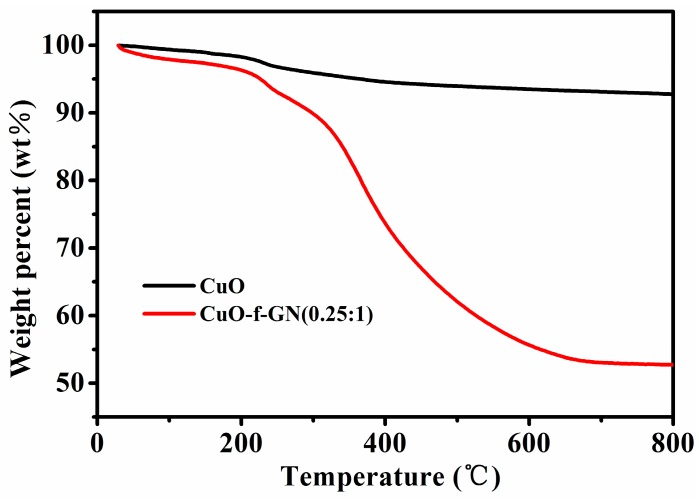
TGA curves of CuO powder and the CuO/f-graphene composite in air.

Figure 3 shows the thermogravimetric analyzer (TGA) results of pure CuO powders and the CuO/f-graphene composite. For the CuO/f-graphene composite, the first step of weight loss (4%) from 30 °C to 200 °C indicates the loss of water in the composite while the second phase of weight loss from 200 °C to 300 °C could be ascribed to the degradation of functional groups of f-graphene. Finally, the graphene were completely decomposed from 300 °C to 800 °C, indicating that the content of CuO is 53 wt % in the CuO/f-graphene composite and the weight ratio of CuO to f-graphene is about 53:47. This result is well matched with the set proportion of raw materials. Thus, it is obvious that the raw materials of the graphene and Cu(OH)_2_ have reacted completely.

**Figure 4:**
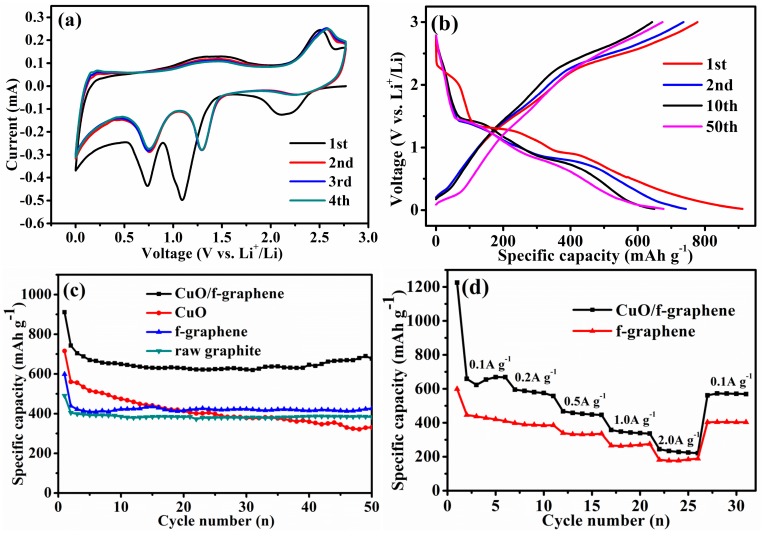
(**a**) The first four CV curves of the CuO/f-graphene composite at a scan rate of 0.1 mV·s^−1^ in the potential range of 0–3.0 V (Li^+^/Li); (**b**) the first, second, tenth, and fiftieth galvanostatic charge/discharge curves of the CuO/f-graphene composite at a current density 100 mA·g^−1^; (**c**) cycling performance of raw graphite, pure CuO, the f-graphene and the CuO/f-graphene composite at a current density of 100 mA·g^−1^; and (**d**) the rate performance of the f-graphene and the CuO/f-graphene composite at various current densities ranging from 0.1 to 2.0 A·g^−1^.

Figure 4a shows the cyclic voltammetry (CV) curves of the CuO/f-graphene composite at a scan rate of 0.1 mV/s. The CV curves were tested with a voltage range from 0 to 3 V to study the electrochemical mechanism. Compared with the CV curves of the CuO/f-graphene composite under the same testing conditions (see Figure S1), in the reduction process, the curves of pure CuO and the CuO/f-graphene composite are composed of three cathodic peaks located at 2.2 V, 1.1 V, and 0.7 V. For the first cycle, the cathodic peak of the CuO/f-graphene composite at 2.2 V could be ascribed to the nonreversible formation of a solid electrolyte interface (SEI) film [32]. The other two reduction peaks could be attributed to a multistep electrochemical reaction, which includes the formation of Cu_2_O phase and the decomposition of Cu_2_O into Cu [23,27,36]. In the oxidation process, the CV curves of the CuO/f-graphene composite reveal three anodic peaks at 0.2, 1.3, and 2.5 V, which could be interpreted as the delithiation of graphene, the reformation of Cu_2_O and CuO, respectively [29,37]. The peaks of the CuO/f-graphene composite are consistent with those of pure CuO (see Figure S1b). The corresponding reaction process is represented as:

CuO + 2Li^+^ + 2e^−^ ↔ Cu + Li_2_O

From the second cycle to the fourth cycle, the CV curves of the CuO/f-graphene composite have higher extent overlapping than the CV curves of pure CuO and other CuO/graphene composite reported before [27], suggesting the good reversibility and structural stability of the CuO/f-graphene composite.

Figure 4b depicts the first, second, tenth, and fiftieth cycles of galvanostatic charge/discharge curves of the CuO/f-graphene composite tested at a current density of 100 mA·g^−1^ with voltage range from 0.01 to 3.0 V. In the first cycle, three voltage plateaus at about 2.0–2.3 V, 1.2–1.5 V, and 0.5–0.8 V are observed. This indicates that during the conversion process, multi-step electrochemical reactions have taken place between CuO and lithium, which correspond to the three cathodic peaks of the CuO/f-graphene composite in the CV curves (see Figure 4a) [38]. Moreover, the highly coincidence of the tenth and fiftieth curves suggest the good stability of the CuO/f-graphene composite.

Figure 4c shows the cycle performance of raw graphite, pure CuO, the f-graphene and the CuO/f-graphene composite at the current density of 100 mA g^−1^. The f-graphene keeps a steady capacity of 426 mAh·g^−1^ after 50 cycles, which is slightly higher than that of raw graphite after the same cycles (383 mAh·g^−1^). It suggests that the introduction of functional groups “–(CH_2_)_5_COOH” can increase the specific surface area of graphene to some extent compared with graphite, thus enhancing the capacity of lithium storage. The pure CuO shows a distinct capacity decline from 716 mAh·g^−1^ to 331 mAh·g^−1^. Contrast this with the f-graphene and pure CuO, where the initial charge and discharge capacities of the CuO/f-graphene composite are 912 mAh·g^−1^ and 778 mAh·g^−1^, respectively. Due to the various irreversible processes mainly including the ineluctable formation of SEI film and electrolyte decomposition, the capacity of the CuO/f-graphene composite has a reasonable loss in the first cycle. After the second cycle, the discharge capacity stabilizes at 677 mAh·g^−1^ during the whole cyclic process. The initial coulomb efficiency of the CuO/f-graphene composite is 85%, which is much higher than previous reports [12,24,26]. And after two cycles, the coulomb efficiency could remain stable at higher than 98% (see Figure S2). Moreover, the capacity loss is negligible, and the specific capacity is higher than other CuO/graphene composites reported before [22,24,26,29]. The CuO/f-graphene composite exhibits good cycle stability and high specific capacity. Compared with pure CuO, the capacity of the composite is more stable during the cyclic test, suggesting that the f-graphene can stabilize and inhibit the volume expansion of CuO. We believe that the introduction of the “–(CH_2_)_5_COOH” functional groups has increased the interlayer spacing of graphene sheets, and the expanded graphene sheets are highly conducive and leave enough space for the copper oxide to be inserted between the f-graphene sheets, which leads to the structural stability after several galvanostatic charge-discharge cycles. In addition, the “–(CH_2_)_5_COOH” groups can provide nucleation sites for the formation of well-dispersed CuO nanocrystals. Figure 4d represents the rate performance of the f-graphene and the CuO/f-graphene composite evaluated at various current densities from 0.1 to 2.0 A·g^−1^. As we can see, the discharge capacities of the CuO/f-graphene composite are 659, 595, 467, 357, and 244 mAh·g^−1^, respectively at current densities of 0.1, 0.2, 0.5, 1.0, and 2.0 A·g^−1^. When the current density is restored to 0.1 A·g^−1^, the discharge capacity goes back to 572 mAh·g^−1^, making it clear that the CuO/f-graphene composite has a decent structural stability. On the other hand, the f-graphene exhibits an excellent stability at the same current densities since the specific capacity varies from 438 mAh·g^−1^ at 0.1 A·g^−1^ to 177 mAh·g^−1^ at 2 A·g^−1^ during the first 25 cycles and recover to 403 mAh·g^−1^ at 0.1 A·g^−1^ in the last five cycles. We may safely draw the conclusion that the stability of the CuO/f-graphene composite is attributed to the f-graphene.

**Figure 5:**
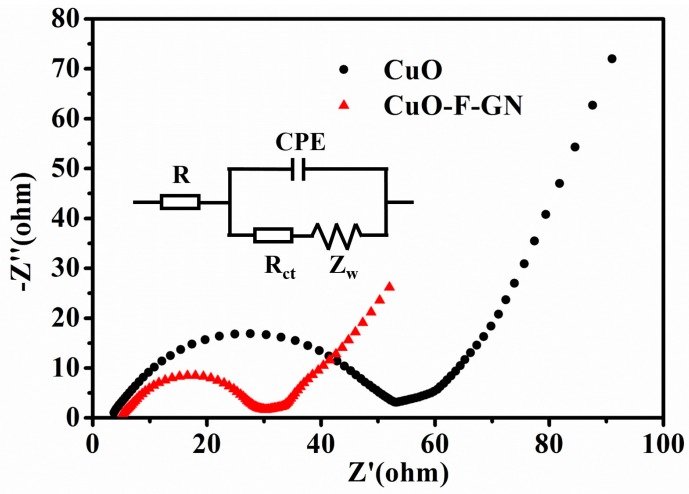
Nyquist plots of pure CuO and the CuO/f-graphene composite electrodes.

Finally, the electrochemical impedance spectroscopy (EIS) was carried out to investigate the kinetics, electric double layers, and diffusion of the electrode reaction process. As depicted in Figure 5, the Nyquist impedance plots of pure CuO and the CuO/f-graphene composite electrodes before cycling performance were tested at 0.5 V with the frequency range from 100 kHz to 10 mHz. The inset presents the equivalent circuit model. R is the Ohmic resistance in the high frequency region and CPE is the constant phase element. Charge-transfer resistance (R_ct_), which engenders between the liquid electrolyte and the active materials, is denoted by the medium-frequency semicircle in the EIS [29]. At low frequencies, the inclined line, called the Warburg impedance (Z_w_), is ascribed to lithium diffusion within the electrodes. Apparently, the CuO/f-graphene composite has a smaller semicircle compared with the pure CuO before cycling, which indicates a lower R_ct_ of the CuO/f-graphene composite than that of the pure CuO. Comparing to the pure CuO, the CuO/f-graphene composite has a better electronic conductivity and charge-transfer performance with the addition of conductive f-graphene [39].

## 4. Conclusions

In summary, the CuO/f-graphene composite material have been successfully synthesized by a one-pot wet chemistry method. The CuO NWs were well-dispersed in the f-graphene sheets with sizes ranging from 300 nm to 500 nm in length and 10 nm to 20 nm in width. As an anode material in LIBs, the CuO/f-graphene composite displays improved electrochemical performance compared to the pure CuO counterpart. It shows a high capacity of 912 mAh·g^−1^ at first cycle at a current density of 100 mA·g^−1^ and a good electrochemical stability with the capacity of 677 mAh·g^−1^ after 50 cycles. The introduction of functional groups “–(CH_2_)_5_COOH” has increased the interlayer spacing of graphene sheets, and the expanded graphene sheets are still conducive and leave enough space for CuO to be inserted between f-graphene sheets, which leads to the structural stability after galvanostatic charge-discharge cycles. In addition, “–(CH_2_)_5_COOH” groups provide nucleation sites for the formation of well-dispersed CuO nanocrystals. Therefore, this method can be a general approach to prepare anode materials for LIBs.

## Supplementary Materials

The following are available online at www.mdpi.com/1996-1944/10/1/72/s1. Figure S1: The first four CV curves of (a) the f-graphene and (b) pure CuO at a scan rate of 0.1 mV·s^−1^ in the potential range of 0–3.0 V (Li^+^/Li). Figure S2: Cycling performance and coulombic efficiency of the CuO/f-graphene composite at a current density of 100 mA·g^−1^.
