# Supplementary material for: Preparation of Advanced CuO Nanowires/Functionalized Graphene Composite Anode Material for Lithium Ion Batteries
Source: Materials (Basel). 2017 Jan 17;10(1):72. doi: 10.3390/ma10010072 (PMC5344618; doi:10.3390/ma10010072)
Supplement: Supplementary file 1 [file materials-10-00072-s001.pdf]

Supplementary Materials: Preparation of Advanced CuO Nanowires/Functionalized Graphene Composite Anode Material for Lithium Ion Batteries

Jin Zhang, Beibei Wang, Jiachen Zhou, Ruoyu Xia, Yingli Chu and Jia Huang

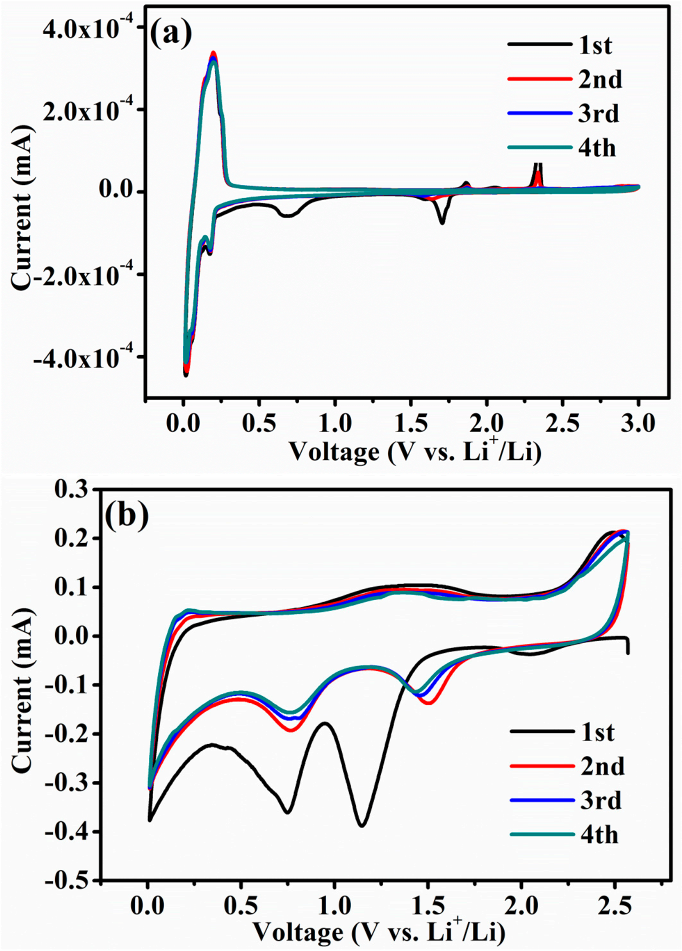

Figure S1. The first four CV curves of (**a**) the f-graphene and (**b**) pure CuO at a scan rate of 0.1 mV·s^{-1} in the potential range of 0–3.0 V (Li$^+$/Li).

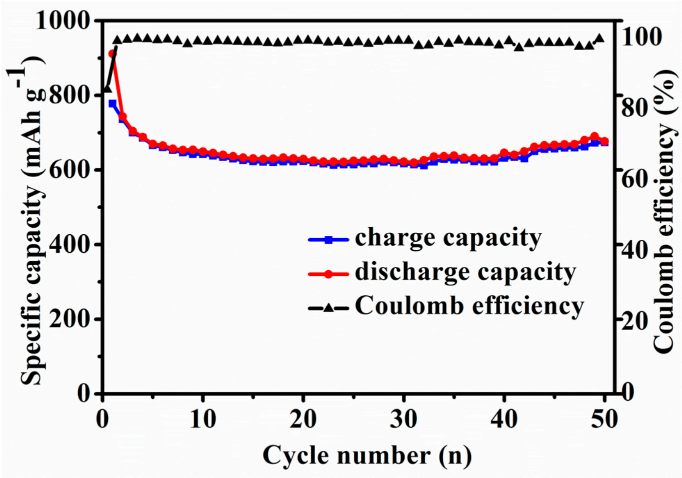

Figure S2. Cycling performance and coulombic efficiency of the CuO/f-graphene composite at a current density of 100 mA·g^{-1}.
